# Upregulation of PD-1 Expression and High sPD-L1 Levels Associated with COVID-19 Severity

**DOI:** 10.1155/2022/9764002

**Published:** 2022-08-01

**Authors:** Danielle Rosa Beserra, Ricardo Wesley Alberca, Anna Claudia Calvielli Castelo Branco, Luana de Mendonça Oliveira, Milena Mary de Souza Andrade, Sarah Cristina Gozzi-Silva, Franciane Mouradian Emidio Teixeira, Tatiana Mina Yendo, Alberto José da Silva Duarte, Maria Notomi Sato

**Affiliations:** ^1^Laboratory of Dermatology and Immunodeficiencies, LIM 56, Tropical Medicine Institute, School of Medicine, University of Sao Paulo, Brazil; ^2^Department of Dermatology, Faculdade de Medicina FMUSP, Universidade de Sao Paulo, 01246-903 Sao Paulo, Brazil

## Abstract

COVID-19 has several mechanisms that can lead to lymphocyte depletion/exhaustion. The checkpoint inhibitor molecule programmed death protein 1 (PD-1) and its programmed death-ligand 1 (PDL-1) play an important role in inhibiting cellular activity as well as the depletion of these cells. In this study, we evaluated PD-1 expression in TCD4+, TCD8+, and CD19+ lymphocytes from SARS-CoV-2-infected patients. A decreased frequency of total lymphocytes and an increased PD-1 expression in TCD4+ and CD19+ lymphocytes were verified in severe/critical COVID-19 patients. In addition, we found a decreased frequency of total monocytes with an increased PD-1 expression on CD14+ monocytes in severe/critical patients in association with the time of infection. Moreover, we observed an increase in sPD-L1 circulant levels associated with the severity of the disease. Overall, these data indicate an important role of the PD-1/PDL-1 axis in COVID-19 and may provide a severity-associated biomarker and therapeutic target during SARS-CoV-2 infection.

## 1. Introduction

In late 2019, the emergence of a novel betacoronavirus was reported in Wuhan, China, causing severe acute respiratory syndrome coronavirus 2 (SARS-CoV-2), which is responsible for the pandemic coronavirus disease 2019 (COVID-19). Immune adaptive response is crucial for the immune response to COVID-19. SARS-CoV-2-specific TCD4+ lymphocytes producing IFN-*γ*, TNF, and IL-2 act in the proinflammatory antiviral response, and follicular T helper cells to induce B cells in affinity maturation and antibody production. In addition, TCD4+ cells cooperate the proliferation of TCD8+ cells [1]. Furthermore, rapid induction of TCD4+ cells during the acute phase of COVID-19 is associated with mild disease and the associated absence with severe or fatal disease [2, 3]. Cytotoxic TCD8+ lymphocytes specific for SARS-CoV-2 also play an important role promoting elimination of the infected cells [4]. In the acute phase of the disease, they show high levels of molecules related to cytotoxic functions, such as IFN-*γ*, granzyme B, perforin, and CD107a, being important for prognosis [5]. B-lymphocytes act mainly in the production of neutralizing antibodies, with the receptor-binding domain portion of the S protein of SARS-CoV-2 as the main target [6, 7].

However, some mechanisms induced by SARS-CoV-2 lead to depletion and exhaustion of lymphocytes in infected patients, due to a direct infection of T-cells via the ACE2 receptor and also the ability of SARS-CoV-2 to lead to atrophy of secondary lymphoid organs, such as the spleen and lymph node [8, 9]. Lymphocyte exhaustion is mainly characterized by the expression of programmed death inhibitory receptor 1 (PD-1) and programmed death ligand 1 (PDL-1) [10], important mediators of central and peripheral immune tolerance and immune exhaustion. High expression levels of PD-1 in T-cells are associated with exhaustion in various viral infections such as HIV, hepatitis B, and hepatitis C [11–13]. In TCD8+ cells, high PD-1 expression can lead to the inability of the cytokine secretion, prevents cell proliferation, and decreases their cytotoxic capacity [14, 15]. In TCD4+ cells, high levels of PD-1 are also correlated with inhibition of cell proliferation and decreased effector functions. Also, it is described that high PD-1 levels, in B-lymphocytes make them able to suppress TCD4+ and TCD8+ lymphocytes via the PD-1/PD-L1-dependent pathway [16]. Monocytes also have an important role in the immune response against COVID-19, where they are recruited by inflammatory mediators to the site of infection and differentiate into inflammatory macrophages and DC-like phenotypes (dendritic cells) to fulfill their effector functions of pro- and anti-inflammatory activities, antigen presentation, and tissue remodeling [17]. The PD-1/PD-L1 axis may also influence the immune response of these cells. In patients with hepatocellular carcinoma, PD-1 expression in monocytes is associated with induction of IL-10 secretion, leading to inhibition of TCD4+ cell activity in HIV patients [18]. Elevated IL-10 levels in COVID-19 patients may be related to the PD-1/PD-L1 axis in the development of acute viral infections and monocyte rearrangement [19]. Furthermore, monocyte depletion in moderate and severe patients was associated with higher frequency of effector memory T-cells expressing the PD-1 depletion marker in COVID-19 [20], suggesting important role of monocytes in the course of the adaptive immune response to SARS-CoV-2.

Taken together, investigation of the modulation of the PD-1/PD-L1 axis in lymphocytes and monocytes in SARS-CoV-2 infection, their soluble forms, and its relationship to the course of the disease becomes necessary.

## 2. Materials and Methods

### 2.1. Ethics Declaration

The study was approved by the Ethics Committee of the Hospital das Clínicas da Faculdade de Medicina da Universidade de São Paulo–HC-FMUSP (CAAE 30800520.7.0000.0068-2020).

### 2.2. Casuistic and Methods

The blood collected with EDTA from patients with COVID-19 was proceeding from the Clinical Division of the Central Laboratory of the Hospital das Clínicas da Faculdade de Medicina da Universidade de São Paulo (HC-FMUSP). The samples were kept at 4°C and analyzed the following day. The study was approved by the Ethics Committee of HC-FMUSP (no. 30800520.7.0000.0068-2020) and was carried out in conformity with the 2013 revision of the Declaration of Helsinki. For inclusion in the study, only samples from patients with a confirmed diagnosis for COVID-19 through the detection of SARS-CoV-2 RNA by reverse transcriptase-polymerase chain reaction were used. Patients over 75 years of age, with neoplasms, transplanted, who are pregnant, and who did not have a positive result for SARS-CoV-2 were excluded from the study. As a healthy control group, individuals without an active SARS-CoV-2 infection and aged over 18 were selected. Patients' samples were collected between May 2020 and September 2021. Patients were divided into mild cases, in which no oxygen therapy or oxygen by mask/nasal prong was required; severe cases, in which they were submitted to noninvasive ventilation; and critical cases, in which they were submitted to invasive mechanical ventilation support, by the time of sample collection, according to *Therapeutic Trial Synopsis*, WHO (2020). In total, samples from 146 individuals were used: 40 of them for phenotypic analysis and 52 for the absolute values of lymphocytes and monocytes; and all samples were used for ELISA assay. The samples of 21 healthy individuals age-matched were used as control.

### 2.3. PD-1 Expression by Flow Cytometry

Peripheral blood samples (100 *μ*L) were incubated with viability marker LIVE/DEAD Fixable Red Dead Cell Stain Kit (Invitrogen, Carlsbad, CA, USA) for 20 minutes at room temperature. After that, it was followed by incubation with surface antibodies to CD3/BV605 (clone SK7), CD4/V500 (clone RPA-T4), CD8/V450 (clone RPA-T8), CD19/PE-Cy7 (clone SJ25C1), CD14/PERCP (clone M*φ*P9), or PD1/APC (clone MIH4) (BD Biosciences, CA, USA) for 30 minutes at room temperature. Next, the samples were fixed with 4% formalin for 15 minutes and the RBCs were lysed with FACS Lysing Reagent (BD Biosciences) for 15 minutes at room temperature. Subsequently, the cells were washed and resuspended in a phosphate buffer, and the acquisition was performed in the flow cytometer LSR Fortessa (BD Biosciences). Approximately 100,000 events per sample were acquired. FlowJo™ software was used to analyze the data obtained.

### 2.4. Measurement of Soluble PD1 and PD-L1

Serum from SARS-CoV-2-infected patients and healthy controls was measured for sPD1 and sPD-L1 by ELISA (Duo-set, R&D, Minneapolis, MN, USA), according to the manufacturer's instructions.

### 2.5. Statistical Analysis

The results were expressed as the median and interquartile range (IQR). The nonparametric Kruskal-Wallis test was used to compare three groups of data. Statistical analyses were performed using Prism 9.0. *P* was considered significant when ≤0.05.

## 3. Results

### 3.1. Demographics of the Cohort Studied

Demographics of COVID-19-infected patients and healthy controls are down in Table 1.

### 3.2. Increased Expression of PD-1 in T and B-Lymphocytes and Monocytes in Patients with COVID-19

**Figure 1:**
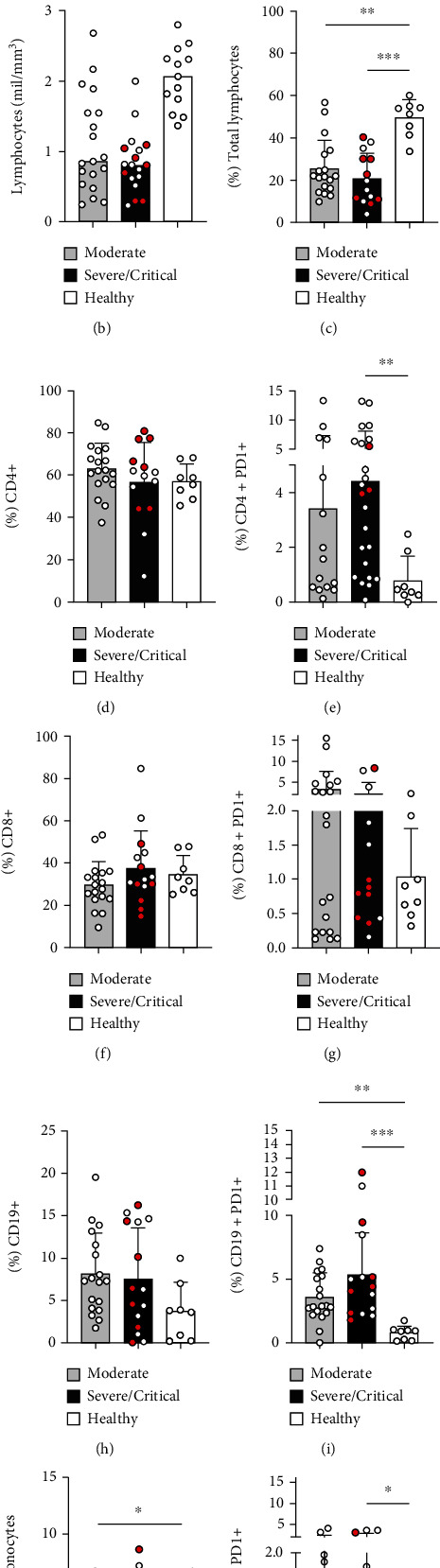
Upregulation of PD-1 in lymphocytes and monocytes of patients with COVID-19. Strategy of the analyses for TCD4+, TCD8+, CD19+, and monocytes expressing PD-1 (a); absolute values of total lymphocytes in patients with moderate (*n* = 19), severe (open symbol, *n* = 16), and critical (red symbol, *n* = 6) disease and healthy control (*n* = 13) (b). Frequency of total lymphocytes (c), CD4+ CD3+ T-cells (d), CD4+ PD-1+ T-cells (e), CD8+ CD3+ T-cells (f), CD8+ PD1+ T-cells (g), CD19+ cells (h), CD19+ PD-1+ cells (i), total monocyte frequency (j), and CD14+ PD-1+ cells (k) assessed by flow cytometry in peripheral blood samples of control subjects (*n* = 8) and patients in moderate (*n* = 18), severe (open symbol, *n* = 7), and critical (red symbol, *n* = 7) clinical status. ^∗^*P* ≤ 0.05, ^∗∗^*P* < 0.01, and ^∗∗∗^*P* < 0.001.

Individuals infected with SARS-CoV-2 showed a decreased frequency of total lymphocytes in all patients (Figure 1(c)); however, lymphopenia was only observed in patients with severe and critical symptoms (Figure 1(b)). Gate strategy of COVID patients is shown in Figure 1(a), and one representative for healthy control is shown in Supplementary Figure 1.

Even though the frequency of TCD4+ does not change in COVID-19 patients compared to healthy control (Figure 1(d)), an increased PD-1 expression on TCD4+ cells was observed in patients with severe and critical symptoms (Figure 1(e)). The frequency of TCD8+ cells does not vary (Figure 1(f)) as well as the PD-1 expression between the analyzed groups (Figure 1(g)).

**Figure 2:**
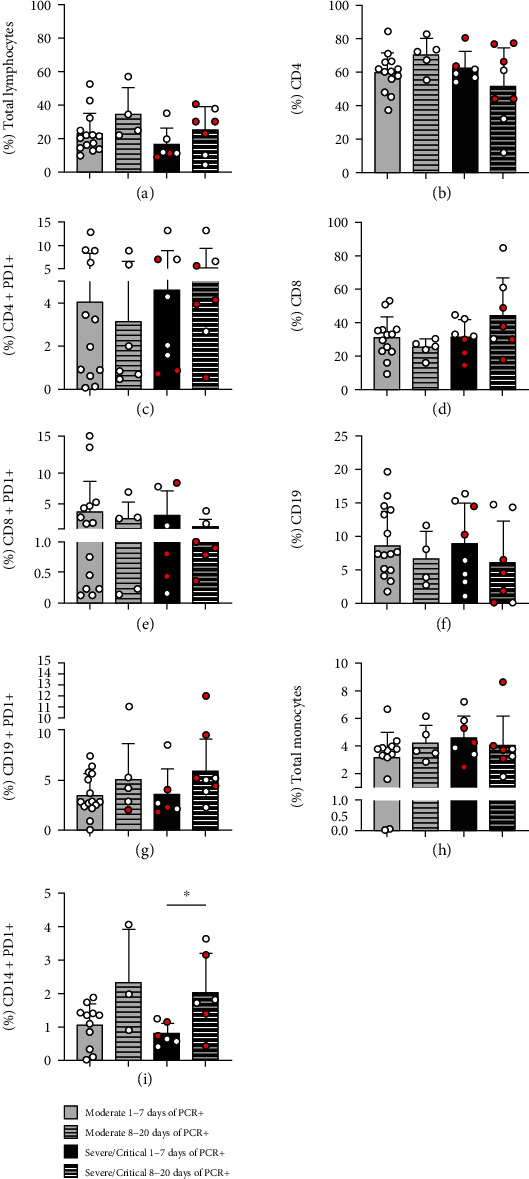
Increased expression of PD-1 on CD14+ monocytes is associated with the duration of SARS-CoV-2 infection. Frequency of total lymphocytes (a), CD4+ CD3+ T-cells (b), CD4+ PD-1+ T-cells (c), CD8+ CD3+ T-cells (d), CD8+ PD1+ T-cells (e), CD19+ cells (f), CD19+ PD-1+ cells (g), total monocyte frequency (h), and CD14+ PD-1+ cells (i) assessed by flow cytometry in peripheral blood samples of control subjects (*n* = 8) and patients in moderate (*n* = 18), severe (open symbol *n* = 7), and critical (red symbol, *n* = 7) clinical status. ^∗^*P* < 0.05.

We also observed an upregulation of PD-1 expression on B-cells in both mild and severe groups (Figure 1(i)). Total monocytes decreased in frequency in moderate patients (Figure 1(j)); meanwhile, PD-1 expression was increased on CD14+ monocytes in patients with severe and critical symptoms (Figure 1(k)). Next, we sorted patients based on the infection time (1-7 days after diagnosis of SARS-CoV-2 by PCR and 8-25 days after), and we observed the percentage of CD14+PD1+ cells increased along the time of infection (Figure 2(i)).

### 3.3. High Levels of sPD-L1 in the Severity Course of COVID-19

**Figure 3:**
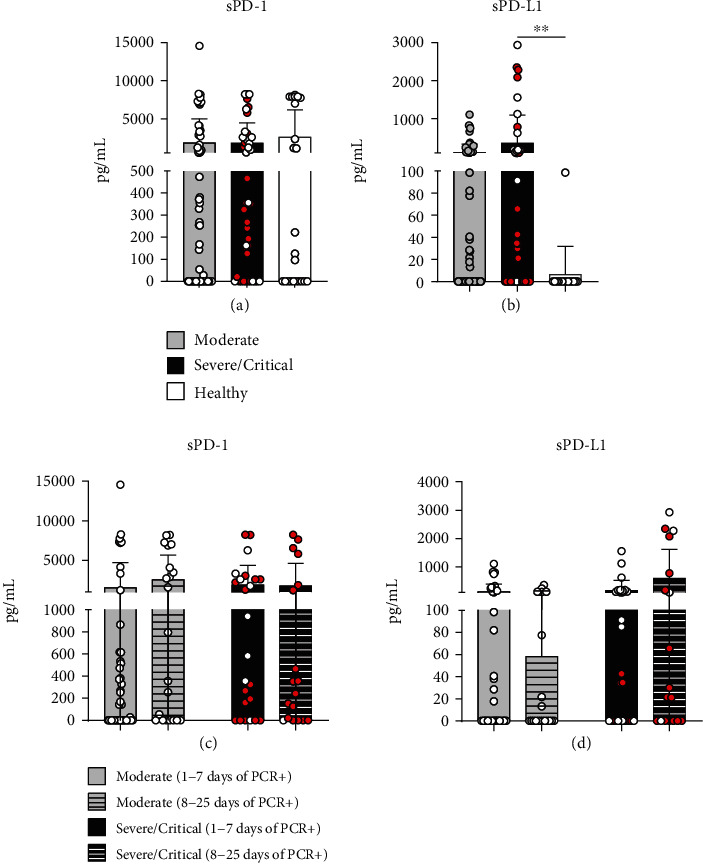
Increased circulant sPDL-1 levels in severe/critical COVID-19 patients. Determination by ELISA of sPD-1 (a) and sPDL-1 (b) in serum from control subjects (*n* = 15) and moderate (*n* = 69), severe (open symbol, *n* = 14), and critical (red symbol, *n* = 29) patients with COVID-19. sPD-1 (c) and sPDL-1 (d) at 1-7 days and 8-25 days of PCR diagnosis for SARS-CoV-2. ^∗∗^*P* < 0.01.

We evaluated the levels of soluble PD-1 and PD-L1 in the serum of 127 patients with 15 control subjects. Similar levels of sPD-1 were detected between the groups (Figure 3(a)); however, there were sPDL-1 increased levels in patients with severe and critical symptoms compared to healthy controls (Figure 3(b)). Moreover, no difference was observed according to sPD-1 (Figure 3(c)) and sPD-L1 (Figure 3(d)) levels along the time of diagnosis for SARS-CoV-2 infection.

## 4. Discussion

In the phenotypic analysis of patients' lymphocytes, we observed a decrease in the frequency of total lymphocytes and lymphopenia only in patients with severe and critical symptoms. In cases of COVID-19, lymphocyte depletion has been described [21, 22]. Several factors may contribute to this picture in affected patients, such as infection of SARS-CoV-2 in T-lymphocytes through the ACE2 receptor [23], involvement of secondary lymphoid organs such as spleen and lymph node [24], and also the COVID-19-characteristic cytokine storm, where elevated levels of TNF-symbol, IL-6, and IL-10 are associated with lymphocyte depletion [25]. Programmed death inhibitory receptor 1 (PD-1) together with its programmed death-ligand 1 (PD-L1) is a key biomarker of lymphocyte depletion [10], where the increased expression of PD-1 blocks signaling pathways such as PI3K/AKT that suppress phosphorylation of PIP2/PIP3 proteins, andthe RAS/MEK/ERK axis, causing the inhibition of lymphocyte proliferation and differentiation. Furthermore, high levels of PD-1/PD-L1 can also suppress glycolysis and direct these cells to the use of lipolysis and fatty acid oxidation for metabolism, slowing it down and potentially contributing to apoptosis [26].

Although the frequency of TCD4+ cells was not altered, we saw an increase in PD-1 expression in patients with severe and critical symptoms. Elevated PD-1 levels in TCD4+ cells are associated with the inhibition of cell proliferation and decreased effector functions [27]. Furthermore, upregulation of PD-1 in T-cells was observed during the progression of symptomatic stages of COVID-19 [28], which contributes to the evolution of the severe form of the disease [23]. In the analysis of TCD8+ cells, we did not observe any difference regarding the frequency or the expression of PD-1; however, one study demonstrated that TCD8+ cells expressing PD-1 may not show dysfunctionality in the acute phase of the disease, suggesting that PD-1 expression in these cells is related to their activation and not necessarily to a depletion picture in COVID-19 [29]. In B-lymphocytes, no difference in frequency was seen either; however, an increased PD-1 expression was observed in all patient groups, with higher expression in patients with severe and critical symptoms. The PD-1/PD-L1 axis has critical regulators of humoral immunity [30]. In diseases such as cancer and HIV, increased PD-1 on B-cells has been associated with disease progression [31, 32]. Furthermore, high levels of PD-1 inhibit B-cell proliferation as well as cytokine production via the B-cell receptor, which positively regulates PD-1 expression [33]. Thus, the exhaustion profile of B-cells may be indicative of the severity of SARS-CoV-2 infection.

Monocytes also play an important role in viral infections and are crucial for a successful immune response [17]. We observed decreased monocyte frequency in patients with moderate symptoms; however, when assessing CD14+PD1+ monocytes, we saw increased PD-1 in patients with severe and critical symptoms, being related to the longer duration of the infection. Increased PD-1 expression in monocytes has been associated with their functional dysfunction during sepsis, suggesting that PD-1 can be used as a marker of the development of monocyte dysfunction [34, 35]. In SARS-CoV-2, infection viral sepsis caused by cytokine storm and microcirculation dysfunction has been associated with the disease severity [36, 37]. With this, increased PD-1 expression in monocytes in COVID-19 may be a possible biomarker for the severity of the infection.

PD-1/PD-L1 proteins also have other forms such as their soluble portion sPD-1 and sPD-L1, important immune-regulatory markers described in various pathologies [38]. Elevated levels of sPD-L1 have been observed in patients with acute pancreatitis, rheumatoid arthritis, chronic hepatitis C, and HIV, associated with the severity and progression of these pathologies [39–42]. We analyzed the levels of sPD-1 and sPD-L1 in patients' serum and observed increased sPD-L1 in patients with severe and critical symptoms, indicating that sPD-L1 may also be related to the severity of COVID-19. Furthermore, high levels of sPD-L1 can induce suppressive effects on activated T-cells through its binding to the PD-1 receptor on the surface of these cells, positively regulating an increased PD-1 expression [43]. The increased sPD-L1 level was also observed in the serum of patients with idiopathic pulmonary fibrosis [44], a pathology related to a higher risk of severe COVID-19 [45].

## 5. Conclusion

Our findings highlight the potential role of the PD-1/PD-L1 axis in COVID-19 and suggest a prognostic role for sPD-L1, labeling it as a biomarker of interest in SARS-CoV-2 infection.

## Figures and Tables

**Table 1:** Demographic data of the COVID-19 patient cohort and control subjects used in the study.

	Healthy (*N* = 21)		Moderate (*N* = 70)		Severe (*N* = 16)		Critical (*N* = 39)	
*Female/male*	11/10		27/43		6/10		18/21	
	Average		Average		Average		Average	
Age (general)	46.42		55.67		49.74		50.13	
Age (F)	45.4		56.6		49.7		59.4	
Age (M)	47.3		54.7		49.7		57	
	*N*	%	*N*	%	*N*	%	*N*	%
*Evolution*								
Medical release	—	—	56	80	15	94	18	46
Death	—	—	12	17	1	6	19	49
Transfer	—	—	2	3	0	0	2	5
*Comorbidities*								
SAH/cardiovascular disorders	—	—	32	28	7	27	26	31
Diabetes mellitus	—	—	19	17	6	23	15	18
Obesity	—	—	8	7	3	11	8	10
Coagulopathies	—	—	1	1	0	0	1	1
Use of alcohol/cigarette	—	—	5	1	0	0	3	4
Serology+HBV HCV	—	—	1	1	0	0	0	0
Liver disorders	—	—	3	3	0	0	2	2
Kidney disorders	—	—	13	12	2	8	5	6
Neurological disorders	—	—	8	7	0	0	3	4
Respiratory disorders	—	—	3	3	1	4	5	6
Metabolic disorders	—	—	13	12	1	4	7	9
Autoimmune disease	—	—	1	1	0	0	0	0
No comorbidity	—	—	5	4	6	23	7	8

Data on age, evolution, and comorbidities of control subjects and COVID-19 patients were classified as moderate, severe, and critical. Legend: M: male; F: female; SC: healthy control; *N*: sample number; SAH: systemic arterial hypertension.

## Data Availability

All datasets generated in this study are included in the article.

## Supplementary Materials

Supplementary Materials Supplementary Figure 1: strategy of the analyses for TCD4+, TCD8+, and CD19+ cells and monocytes expressing PD-1 from healthy controls.
