# Supplementary figures and images for: Upregulation of PD-1 Expression and High sPD-L1 Levels Associated with COVID-19 Severity
Source: J Immunol Res. 2022 Aug 1;2022:9764002. doi: 10.1155/2022/9764002 (PMC9375698; doi:10.1155/2022/9764002)

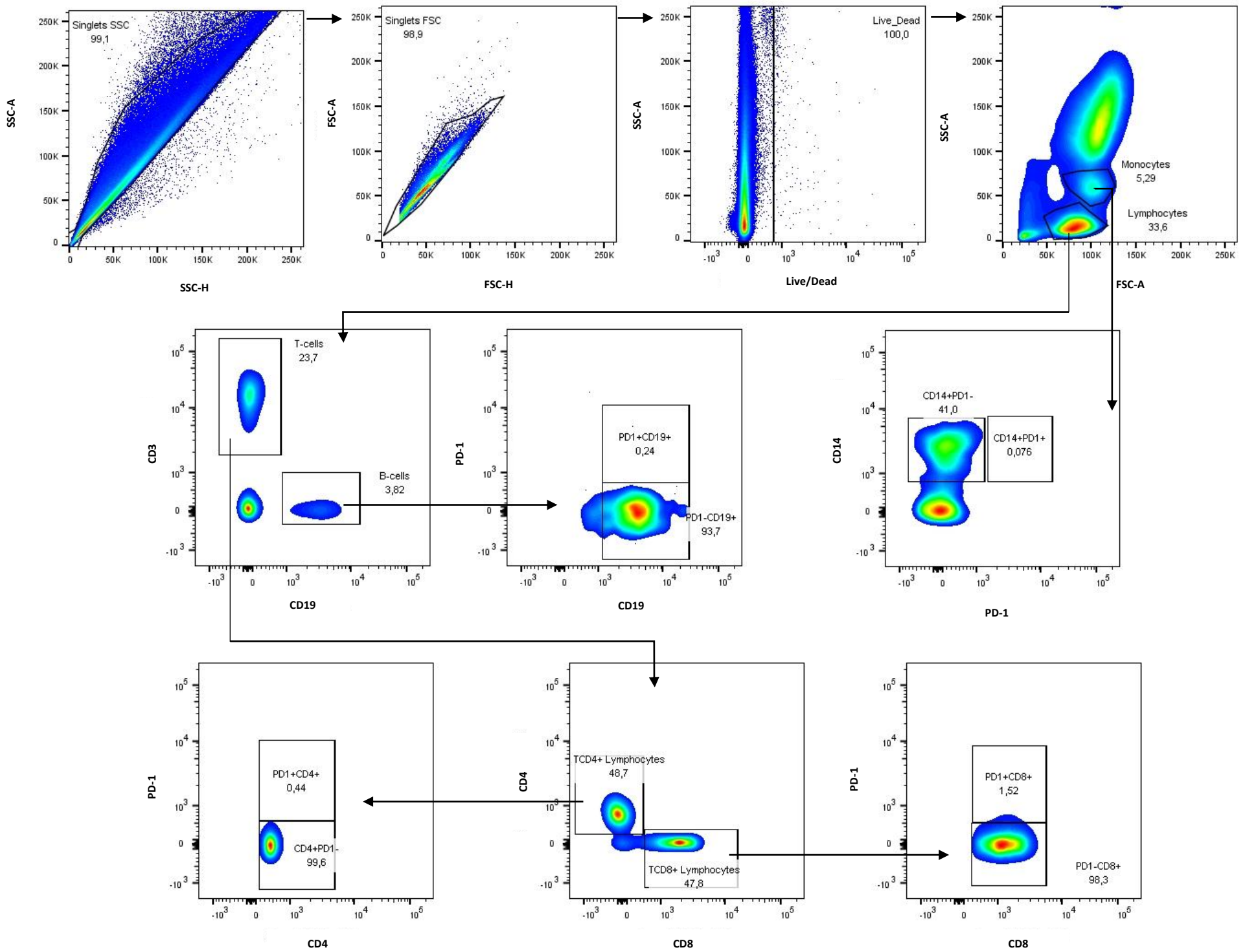

Supplement: Supplementary Materials — Supplementary Figure 1: strategy of the analyses for TCD4+, TCD8+, and CD19+ cells and monocytes expressing PD-1 from healthy controls. [file 9764002.f1.pdf]
